# Functional evaluation with microperimetry in large idiopathic macular holes treated by a free internal limiting membrane flap tamponade technique

**DOI:** 10.1186/s12886-020-01573-z

**Published:** 2020-07-23

**Authors:** Peirong Huang, Hong Wang, Fenge Chen, Jieqiong Chen, Yifan Hu, Junran Sun, Jingyang Feng, Hong Zhu

**Affiliations:** 1 Department of Ophthalmology, Shanghai General Hospital (Shanghai First People’s Hospital), Shanghai Jiao Tong University School of Medicine, No.100 Hai Ning Road, Shanghai, 200080 China; 2 Shanghai Key Laboratory of Fundus Disease, Shanghai, China; 3 Shanghai Engineering Center for Visual Science and Photomedicine, Shanghai, China

**Keywords:** Idiopathic macular hole, Microperimetry, Internal limiting membrane, Foveal sensitivity, Fixation stability

## Abstract

**Background:**

Free internal limiting membrane (ILM) flap tamponade technique is an alternative choice for treating large idiopathic macular holes (IMHs). However, the functional recovery related to this surgical approach is not well-characterized. This study aimed to evaluate morphological and microperimetric outcomes 6 months after free ILM flap tamponade technique for large IMHs.

**Methods:**

Twenty-two patients (22 eyes) with large IMHs (minimal diameter > 400 μm) were retrospectively enrolled in this study. All patients underwent 23-gauge pars plana vitrectomy with ILM peeling and free ILM flap tamponade procedures. Snellen best-corrected visual acuity (BCVA), optical coherence tomography (OCT), and MP-1 microperimetry were measured at baseline and 6 months after surgery. Associations of postoperative BCVA with retinal sensitivity were detected.

**Results:**

Macular hole closure was achieved in 21 eyes (95.5%). Dislodgement of free ILM flap was found in non-closed eye. Mean logMAR BCVA improved from 1.10 ± 0.33 at baseline to 0.67 ± 0.32 at 6 months postoperatively (*P* < 0.001). The mean overall macular sensitivity and foveal fixation stability increased respectively from 8.58 ± 3.05 dB and 65.64 ± 17.28% before surgery to 11.55 ± 2.72 dB and 78.59 ± 13.00% at 6 months after surgery (*P* < 0.001). The mean change in foveal sensitivity (within 2°) was significantly greater than the change achieved for peri-foveal sensitivity (2° to 10°) by 1.50 ± 2.62 dB (*P* = 0.014). Linear regression analysis showed that postoperative logMAR BCVA was significantly associated with duration of symptom (*B* = 0.063, *P* = 0.001), preoperative logMAR BCVA (*B* = 0.770, *P* = 0.000), preoperative peri-foveal (*B* = − 0.065, *P* = 0.000) and foveal sensitivity (*B* = − 0.129, *P* = 0.000). Moreover, multiple regression model revealed that preoperative foveal sensitivity was independently associated with postoperative logMAR BCVA (*B* = − 0.430, *P* = 0.040).

**Conclusions:**

Vitrectomy combined with ILM peeling and free ILM flap tamponade technique results in effective morphological and functional recovery for large IMHs. Preoperative foveal sensitivity might be a prognostic indicator for postoperative BCVA.

## Background

Idiopathic macular holes (IMHs) always affect middle-aged and elderly people and cause severe central vision impairment [1, 2]. Standard vitrectomy combined with the internal limiting membrane (ILM) peeling technique has been reported to achieve an excellent closure rate for macular holes with diameters smaller than 400 μm [3]. However, large macular holes (minimum diameter > 400 μm) are less likely to close after a classic vitrectomy [4, 5]. Although, several modified techniques have been introduced [6–8], the surgical procedures for large, recurrent or refractory macular holes have not yet reached a common consensus.

The free ILM flap tamponade or insertion technique is an effective and relative handy operation for large macular holes [9, 10]. This procedure is easily conducted and could be a remedy in case inverted ILM flap cracks accidently occur or ILM around the hole has already been removed. Although satisfactory anatomical results have been achieved, the effect of tamponade technique on retinal functional restoration is controversial. It is considered that the tamponade of ILM flap inside the macular hole may cause ellipsoid zone defects and affect neural retina recovery [11]. Thus, a deeper investigation of macular function after ILM tamponade surgery is needed and has important clinical significance.

Since best-corrected visual acuity (BCVA) reflects basic visual function, most studies related to ILM tamponade surgery mainly focused on visual acuity measurements. Very few researches have reported the foveal sensitivity when the free ILM flap tamponade has been used to treat large IMHs. Microperimetry, which comprises an automatic real-time tracking system to compensate eye movements, offers sub-regional retinal sensitivity and fixation assessments in precise location on fundus imaging [12]. Microperimetry has already been shown to achieve good efficacy and provide more detailed information of macular function in normal people or patients with macular disorders [13]. Moreover, it has been found that retinal sensitivity is more related than BCVA to the reading ability in patients with fundus diseases [14, 15]. Therefore, the purpose of this present study was to evaluate not only morphology but also retinal sensitivity and fixation stability before and at 6 months after vitrectomy combined with ILM peeling and free ILM flap tamponade procedures for large IMHs.

## Methods

This retrospective case-series study included 22 patients (22 eyes) with large IMHs (Stage III or Stage IV, minimum diameter > 400 um) who underwent 23-gauge pars plana vitrectomy (PPV) with ILM peeling, free ILM tamponade and fluid-air exchange at Shanghai General Hospital from November 2016 to December 2017. All patients were followed up for at least 6 months after surgery. This study was approved by the ethics committee of Shanghai General Hospital and adhered to the tenets of the Declaration of Helsinki.

All patients underwent comprehensive ophthalmological examinations before and at 6 months after surgery. The examinations included Snellen BCVA, optical coherence tomography (OCT, Heidelberg, Germany), and MP-1 microperimetry (NIDEK, NAVIS Software 3.6.4, Gamagori, Japan).

### Patient eligibility

Inclusion criteria: 1) patients with idiopathic macular holes; 2) minimal hole diameter greater than 400 μm; and 3) follow-up longer than 6 months.

Exclusion criteria: 1) any other retinal disease; 2) high myopia with a refractive error of more than − 6.00 dioptres or an axial length longer than 26 mm; 3) server cataract; 4) a history of previous vitreoretinal surgery; and 5) severe systemic disease.

### Microperimetry evaluation

MP-1 microperimetry was conducted in a dark room. After pupil dilation (1% tropicamide), microperimetry was performed in the eye, and the contralateral eye was patched. Macular sensitivity was tested in a program implemented in Macular 10° that consisted of 40 points arranged in 3 concentric circles (2°, 6° and 10°). Goldmann III stimuli (10 cd/m^2^) randomly presented for a duration of 200 milliseconds on a 1.27 cd/m^2^ background. Sensitivity (dB, decibels) was assessed using a 4–2 staircase strategy ranging from 0 dB to 20 dB in 2 dB steps. The mean overall macular sensitivity was calculated as the average recognized threshold of 40 points within 10° of the centre. The mean foveal sensitivity and mean peri-foveal sensitivity were indicated as points within 2° of the centre and points within 2° to 10°. Quantitative foveal fixation stability was measured as the percentage of fixation points within 2° of the centre.

### Surgical procedure

A standard 3-port 23-gauged PPV was conducted by the same surgeon using a Constellation device (Alcon, Fort Worth, TX). After core vitrectomy, the posterior hyaloid was removed from the retina with the assistance of triamcinolone acetonide (TA, 2.5 mg/ml), and a complete vitrectomy was performed at the peripheral vitreous base. Any macular epiretinal membrane was removed if present. Then, indocyanine green (ICG) solution (1.5 mg/ml) was applied to stain the ILM around the macular hole within the arcade. The ILM was peeled around the hole to a diameter of approximately three optic disk diameters. Suitable pieces of ILM were chosen by the surgeon and placed inside the macular hole. The position of free ILM flap was retained by tucking and trapping the edge of pieces into the macular hole using intraocular forceps. The size of the ILM used for tamponade in each patient depended on the macular hole diameter. After confirmation that the transplanted ILM flap was inside the macular hole, abundant fluid-air exchange was slowly performed using sterilized air. If there was air leaking through any of the three incisions, a transscleral suture was applied. All patients were required to maintain a face-down position for at least 3 days after surgery.

### Statistical analysis

The BCVA was converted to the logarithm of the minimum angle of resolution (logMAR). Changes in logMAR BCVA between before and after surgery were analysed using the Wilcoxon signed-rank test. Pre- and postoperative retinal sensitivities were analysed using the paired t-test. Linear regression analysis was performed to investigate the relationship between postoperative logMAR BCVA and other simple variables such as retinal sensitivity. Multivariate regression analysis with backward stepwise method was performed to assess the influence of variables on postoperative logMAR BCVA. All statistical analyses were performed using SPSS 21.0 for Windows. A *P* value of < 0.05 was considered to be statistically significant.

## Results

### Baseline characteristics

Twenty-two eyes of 22 patients (5 men and 17 women) with Stage III (*n* = 16, 72.7%) and Stage IV (*n* = 6, 27.3%) IMHs were enrolled and analysed in this study. Table 1 shows the patient characteristics and baseline clinical data. The average age of the patients was 62.41 ± 8.13 years old (range: 43 ~ 78 years old). The mean baseline logMAR BCVA of the study eye was 1.10 ± 0.33. The mean minimum diameter (MD) of IMHs was 592.12 ± 165.76 μm. The mean basal diameter of IMHs was 1008.04 ± 262.72 μm. At the time of surgery, 6 eyes (27.3%) were pseudophakic.

**Table 1:** Baseline characteristics and 6-month postoperative results of all cases (*n* = 22)

Characteristics	Baseline (n = 22)	6-month (n = 22)	***P***-value
**Age (years)**			
Mean ± SD	62.41 ± 8.13	–	–
Range	43 ~ 78	–	–
**Gender**			
Male, n (%)	5 (22.7)	–	–
Female, n (%)	17 (77.3)	–	–
**Side**			
Right, n (%)	12 (54.6)	–	–
Left, n (%)	10 (45.4)	–	–
**Duration of symptoms (months)**			
Mean ± SD	7.77 ± 3.09	–	–
Range	3 ~ 14	–	–
**IMH stage**			
Stage 3	16	–	–
Stage 4	6	–	–
**MD of IMH (μm)**	592.12 ± 165.76	–	–
**BD of IMH (μm)**	1008.04 ± 262.72	–	–
BCVA (logMAR)*			
Mean ± SD	1.10 ± 0.33	0.67 ± 0.32	*P* < 0.001
**Macular sensitivity (dB)**^**#**^			
Overall (within 10^o^)	8.58 ± 3.05	11.55 ± 2.72	t = 7.176, *P* < 0.001
Peri-foveal (2^o^ to 10^o^)	9.88 ± 3.48	13.07 ± 2.22	t = 5.465, *P* < 0.001
Foveal (within 2^o^)	3.39 ± 1.98	7.55 ± 2.92	t = 10.700, *P* < 0.001
**Foveal fixation (%)**^**#**^	65.64 ± 17.28	78.59 ± 13.00	t = 7.125, *P* < 0.001

IMH = idiopathic macular hole, MD = minimum diameter, BD = basal diameter, BCVA = best corrected visual acuity, logMAR = logarithm of the minimum angle of resolution

^*^Wilcoxon signed rank test, compared with baseline; ^#^paired t test, compared with baseline

### Morphologic and visual outcome

**Fig. 1:**
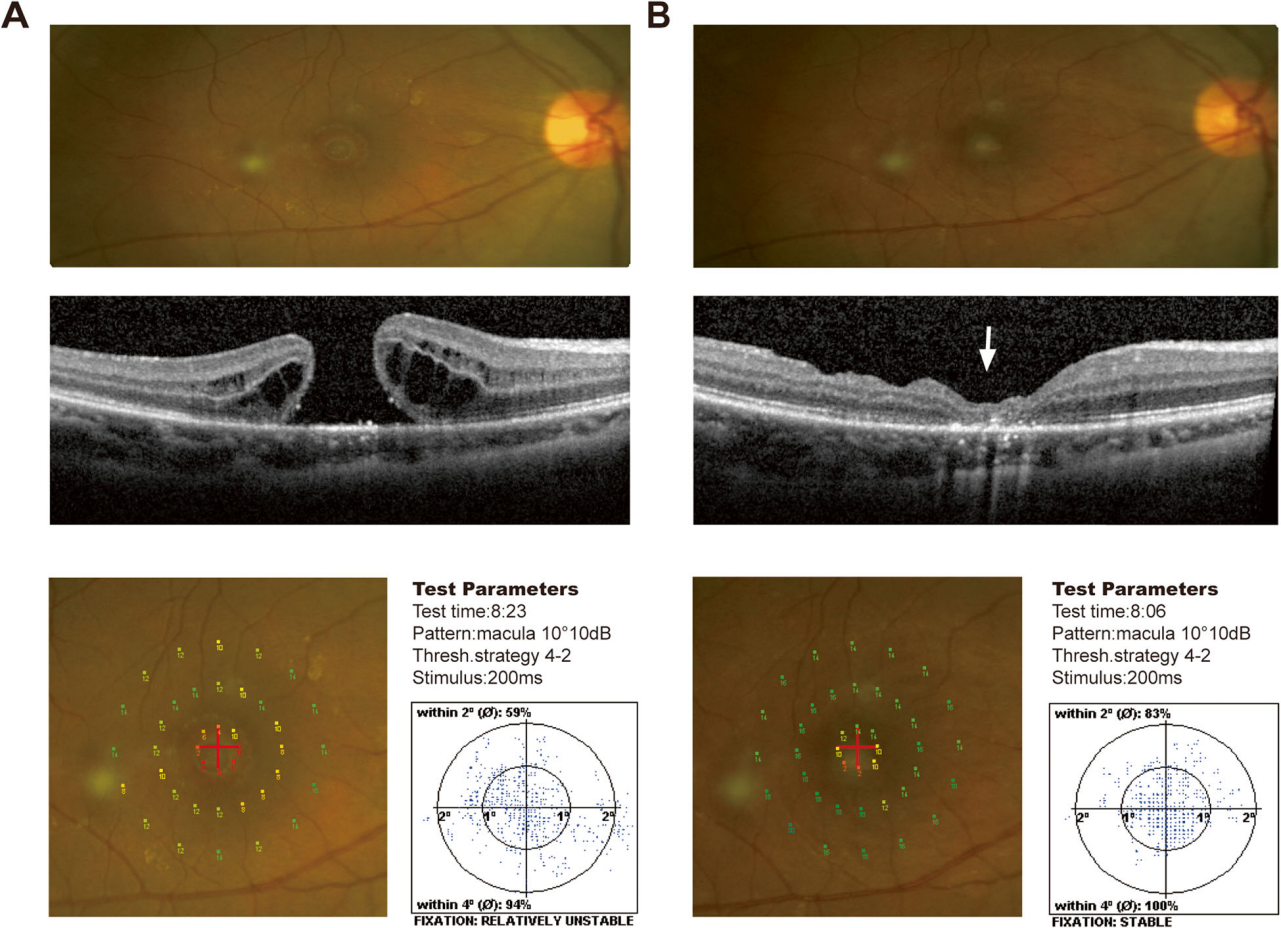
Results of a large IMH patient at baseline (**a**) and 6 months after ILM tamponade surgery (**b**). SD-OCT showed the minimal diameter of the macular hole before surgery was 607 μm. The macular hole was close and filled with amorphous tissues at 6 months after surgery (white arrow). Microperimetry showed the improvement of overall macular sensitivity, especially foveal sensitivity, at 6 months postoperatively. The foveal fixation upgraded from relative unstable to stable. The fixation stability within 2° increase from 59% at baseline to 83% at 6 months postoperatively

At 6 months after surgery, the macular hole was closed in 21 eyes (95.5%). SD-OCT images showed that the fovea was filled with amorphous tissues (Fig. 1). In one unclosed eye, the macular hole showed a flat and open configuration. The mean study eye logMAR BCVA improved significantly from 1.10 ± 0.33 before surgery to 0.67 ± 0.32 at 6 months postoperatively (*P* < 0.001). An improvement of at least two lines was observed at the 6-month follow-up in 16 eyes (72.7%). No complications, including high intraocular pressure, endophthalmitis, or retinal detachment, were observed in any cases at the last follow-up.

### Microperimetry outcome

Microperimetry was assessed preoperatively and 6 months postoperatively in the 22 patients. The mean overall macular sensitivity within 10° of the centre was 8.58 ± 3.05 dB before surgery and 11.55 ± 2.72 dB at 6 months after surgery (t = 7.176, *P* < 0.001). The mean foveal sensitivity within 2° of the centre improved from 3.39 ± 1.98 dB preoperatively to 7.55 ± 2.92 dB postoperatively (t = 10.700, *P* < 0.001). The mean peri-foveal sensitivity within 2° to 10° improved from 9.88 ± 3.48 dB to 13.07 ± 2.22 dB (t = 5.465, *P* < 0.001). The mean change in foveal retinal sensitivity (within 2°) was significantly greater than the mean change in peri-foveal retinal sensitivity (2° to 10°) by 1.50 ± 2.62 dB (t = 2.686, *P* = 0.014).

The foveal fixation stability, calculated as the percent of fixation points within 2° of the centre, had increased in 20 eyes (90.9%) and remained equal in 2 eyes (9.1%) at 6 months postoperatively. The average foveal fixation stability was 65.64 ± 17.28% before surgery and 78.59 ± 13.00% at 6 months after surgery (t = 7.125, *P* < 0.001).

### Association outcome

The linear regression analysis of simple variables revealed that 6-month postoperative logMAR BCVA was significantly associated with duration of symptom (*B* = 0.063, *P* = 0.001), preoperative logMAR BCVA (*B* = 0.770, *P* = 0.000), preoperative peri-foveal (*B* = − 0.065, *P* = 0.000) and foveal sensitivity (*B* = − 0.129, *P* = 0.000). Furthermore, multiple regression model with backward stepwise method showed that preoperative foveal sensitivity was independently associated with postoperative logMAR BCVA (*B* = − 0.430, *P* = 0.040) (Table 2).

**Table 2:** Linear regression analysis of 6-month postoperative logMAR BCVA with single variables (A); multiple linear regression model with backward stepwise method (B)

Variables	(A)				(B)^*^			
	*B*	*Se (B)*	*P*-value	95% CI	*B*	*Se (B)*	*P*-value	95% CI
**Age (years)**	0.000	0.007	0.972	−0.015 to 0.015	− 0.004	0.003	0.253	−0.011 to 0.003
**Gender**	0.013	0.157	0.935	−0.315 to 0.340	−0.079	0.081	0.342	−0.252 to 0.093
**Duration of symptom (months)**	0.063	0.017	0.001	0.028 to 0.098	0.011	0.014	0.455	−0.020 to 0.042
**MD of IMH (**μm**)**	0.001	0.000	0.164	0.000 to 0.001	0.000	0.000	0.591	−0.001 to 0.000
**Pre logMAR BCVA**	0.770	0.110	0.000	0.541 to 0.999	0.290	0.188	0.145	−0.112 to 0.692
**Pre macular sensitivity (dB)**								
Peri-foveal (2^o^ to 10^o^)	−0.065	0.013	0.000	−0.091 to − 0.038	−0.242	0.146	0.146	−0.050 to 0.008
Foveal (within 2^o^)	−0.129	0.018	0.000	−0.166 to − 0.091	−0.430	0.040	0.040	−0.127 to − 0.003

Se (B) = standard error of B coefficient, MD = minimum diameter, IMH = idiopathic macular hole, BCVA = best corrected visual acuity, logMAR = logarithm of the minimum angle of resolution, pre = preoperative

*Adjusted R^2^ for variables listed: 0.786

## Discussion

Our study supports the efficacy of the free ILM flap tamponade technique for the treatment of eyes with large IMHs. At 6 months postoperatively, we found that 1) a high closure rate and significant BCVA improvement were achieved; 2) the mean overall macular sensitivity had significantly increased, with the foveal area within 2° of centre mostly increased; 3) foveal fixation stability had improved; and 4) preoperative foveal sensitivity was independently associated with postoperative logMAR BCVA.

With the modern standard of pars plana vitrectomy performed with the ILM peeling and gas tamponade, small IMHs achieved a good anatomical result. However, relatively low closure rates (50 to 80%) and unsatisfactory functional outcomes still existed in large macular holes (MD > 400 μm) [16]. Currently, several updated techniques, including the inverted ILM flap covering technique and the ILM flap insertion manoeuvre, have been reported to achieve a satisfactory closure rate [17, 18]. The ILM insertion technique, which uses free internal limiting membrane flap tamponade in macular holes, was primarily employed by Morizane et al. [19] for refractory or recurring eyes with macular holes. Based on optimal surgical outcomes, De Novelli et al. [20] employed this technique in initially large macular holes or chronic macular hole surgery with a high successful rate. Compared with inverted ILM flap, this tamponade technique has superiority when the foveal ILM has already been peeled or if the inverted ILM flap detaches from the edge of macular hole during the surgery. This technique could be used in large macular holes or as an alternative remedy in the above circumstances. The crucial point of this surgical procedure is to prevent the dislodging of the free ILM flap from the hole. In our surgery, we used intraocular forceps to trap the margin of free ILM sheet under the edge of macular hole and performed very slow fluid-air exchange to preserve the pieces inside the macular hole. Additionally, in another study, deuteroxide was injected over the free flap for stabilization [21]. In our study, 21 eyes (95.5%) achieved hole closures without loss of dislodging of the ILM flaps. The fovea filled with amorphous tissues represented a relatively normal contour. Comparatively, a former study revealed a similar closure rate (96.0%) in large macular hole patients who underwent inverted ILM flap surgery [22]. Our study suggested a relatively satisfying option for surgeons.

The recovery of visual acuity after flap tamponade technique was confirmed in our study. Our baseline and 6-month-postoprerative BCVA is comparable with a previous large-size multicentre research related to inverted ILM flap technique [23]. The research showed that the mean logMAR BCVA improved from preoperative 1.05 ± 0.31 to 0.57 ± 0.33 at 6 months postoperatively in extra-large macular holes (MD > 550 μm), and from 1.14 ± 0.36 to 0.73 ± 0.35 in super extra-large macular holes (MD > 700 μm). The relative worse baseline and postoperative BCVA in our study might attribute to the lens status and duration of symptom. As in previous research, cataract surgery was performed simultaneously with vitrectomy. The relatively long duration of symptom in our study (7.77 ± 3.09 months) might also contribute to a more challenging situation of disease and a worse pre- and post-operative BCVA.

In addition to improving postoperative BCVA and favourable closure rates, this study introduced microperimetry for the evaluation of retinal function before and after ILM tamponade surgery for large macular holes. Although BCVA is recognized as a basic assessment for foveal function, it may underestimate functional recovery in patients who underwent macular holes surgeries because of possible change of fixation point [24]. Microperimetry has been applied to measure a point to point retinal sensitivity and fixation in various macular diseases [25, 26]. A previous study about relatively smaller macular holes and recurrent macular holes evaluated the visual outcomes 1 year after ILM flap transposition. They suggested that besides BCVA, retinal sensitivity provides more specific functional information about the macula and better reflects the visual restoration after macular surgery [27]. There are potential concerns that the insertion of the ILM inside the macular hole may hinder the recovery of retinal function. For example, the ILM sheet may form a block that prevents the healing of the neurosensory retina and the reconstruction of photoreceptors, or the RPE may be damaged during the insertion procedure. In the present study, we recorded foveal (with 2°) and peri-foveal (2° to 10°) macular sensitivity separately. According to the equipment and fundus structure, 1° converts to 250 μm. The detection range of foveal sensitivity (within 2°) equals 500 μm, which represents the function over the area of the macular hole. We observed that the mean overall macular sensitivity increased from 8.58 ± 3.05 preoperatively to 11.55 ± 2.72 at 6 months after surgery. Additionally, the mean change in foveal sensitivity (4.17 ± 1.83 dB) was greater than the change in peri-foveal sensitivity (2.67 ± 2.29 dB). These results reveal that the improvement in microperimetry after surgery mainly occurred in the fovea, where the macular hole is located, and that the ILM tamponade technique may have an advantage in retinal function recovery. The exact mechanism of ILM flap tamponade in foveal functional recovery requires further investigation. One hypothesis is that the ILM pieces filled into the macular hole contain Müller cells and form a scaffold that stimulates long-lasting glial cell proliferation. Serveal previous studies also observed similar results after different macular hole surgery. Sborgia et al. reported that both central macular sensitivity (within 4°) and macular sensitivity (within 12°) significantly improved in large macular hole after treatment of inverted ILM flap technique [28]. Wang et al. recorded increase of macular hole sensitivity after successful ILM peeling surgery [29].

Fixation is another important macular functional parameter that reflects the resolution of scotoma after surgery in Microperimeter-1. When macular disease occurs, fixation spot could be spontaneously relocated outside the foveal range. A study illustrated that patients with IMHs always had paracentral fixation, which was located 2° outside of the centre at the margin of the macular hole before surgery. After vitreoretinal surgery, an acceptable percentage of patients obtained stable or relative central fixation [30]. In our current study, similar stable fixation recovery was achieved. The average foveal fixation stability within 2° of the centre increased from 65.64 ± 17.28% preoperatively to 78.59 ± 13.00%, and 20 eyes (90.9%) showed improved fixation stability at 6 months after surgery. It is reported that the closure of macular hole could result in restoration and reorganization of fixation [31]. These results suggest that macular function partially recovered after ILM tamponade in the macular hole.

Several studies have investigated possible predictors for visual acuity prognosis of IMHs surgery such as ellipsoid zone defect area [32], macular hole closure index (MHCI) [33] or diameter of macular holes [34]. Our results revealed that mean preoperative foveal sensitivity (within 2°) was independently associated with postoperative logMAR BCVA at 6 months after ILM flap tamponade for larger IMHs, whereas preoperative BCVA and mean preoperative peri-foveal sensitivity (2° to 10°) were less predictive factors. Since foveal sensitivity (within 2°) represents the function over the area of the macular hole, a possible explanation for this finding may be attributed to the fact that eyes with better foveal sensitivity before surgery may reflect less damage of neurosensory retina within the area of macular hole and may therefore have a better recovery and visual outcomes after surgery. A previous report focused on macular hole surgery also found a closer connection between preoperative central macular sensitivity and postoperative visual recovery, which was consistent with our results on large IMHs [35]. These data indicate that preoperative foveal sensitivity is a better prognostic indicator than preoperative BCVA for IMHs.

## Conclusions

Despite some limitations exist including a relatively small sample, relatively short follow-ups and lack of controls, this study reported that improvement in retinal sensitivity and fixation stability was achieved in large IMHs undergoing the free ILM flap tamponade technique and that preoperative foveal sensitivity may be an effective predictor of postoperative BCVA. In summary, microperimetry is a more precise measurement technique than BCVA for surgeons to follow-up retinal function in IMHs. ILM flap tamponade technique is helpful not only for macular hole closure but also for macular function recovery.

## Supplementary information

**Additional file 1.**

## Data Availability

The datasets created during and/or analyzed during the current study available from the corresponding author on reasonable request.

## Abbreviations

IMHs: Idiopathic macular holes
ILM: Internal limiting membrane
BCVA: Best-corrected visual acuity
OCT: Optical coherence tomography
PPV: Pars plana vitrectomy
dB: Decibels
logMAR: Logarithm of the minimum angle of resolution
MD: Minimum diameter
